# RootPainter: deep learning segmentation of biological images with corrective annotation

**DOI:** 10.1111/nph.18387

**Published:** 2022-08-10

**Authors:** Abraham George Smith, Eusun Han, Jens Petersen, Niels Alvin Faircloth Olsen, Christian Giese, Miriam Athmann, Dorte Bodin Dresbøll, Kristian Thorup‐Kristensen

**Affiliations:** ^1^ Department of Plant and Environmental Science University of Copenhagen Højbakkegårds Alle 13 Tåstrup 2630 Denmark; ^2^ Department of Computer Science University of Copenhagen Universitetsparken 1 2100 Copenhagen Denmark; ^3^ CSIRO Agriculture and Food PO Box 1700 Canberra ACT 2601 Australia; ^4^ Department of Agroecology and Organic Farming University of Bonn Regina‐Pacis‐Weg 3 53113 Bonn Germany; ^5^ Department of Organic Farming and Plant Production University of Kassel Nordbahnhofstr. 1a D‐37213 Witzenhausen Germany

**Keywords:** biopore, deep learning, GUI, interactive machine learning, phenotyping, rhizotron, root nodule, segmentation

## Abstract

- Convolutional neural networks (CNNs) are a powerful tool for plant image analysis, but challenges remain in making them more accessible to researchers without a machine‐learning background. We present RootPainter, an open‐source graphical user interface based software tool for the rapid training of deep neural networks for use in biological image analysis.
- We evaluate RootPainter by training models for root length extraction from chicory (*Cichorium intybus* L.) roots in soil, biopore counting, and root nodule counting. We also compare dense annotations with corrective ones that are added during the training process based on the weaknesses of the current model.
- Five out of six times the models trained using RootPainter with corrective annotations created within 2 h produced measurements strongly correlating with manual measurements. Model accuracy had a significant correlation with annotation duration, indicating further improvements could be obtained with extended annotation.
- Our results show that a deep‐learning model can be trained to a high accuracy for the three respective datasets of varying target objects, background, and image quality with < 2 h of annotation time. They indicate that, when using RootPainter, for many datasets it is possible to annotate, train, and complete data processing within 1 d.

## Introduction

Plant research is important because we need to find ways to feed a growing population whilst limiting damage to the environment (Lynch, 2007). Plant studies often involve the measurement of traits from images, which may be used in phenotyping for genome‐wide association studies (Rebolledo *et al*., 2016), comparing cultivars for traditional breeding (Walter *et al*., 2019), or testing a hypothesis related to plant physiology (Rasmussen *et al*., 2020). Plant image analysis has been identified as a bottleneck in plant research (Minervini *et al*., 2015). A variety of software exists to quantify plant images (Lobet *et al*., 2013) but is typically limited to a specific type of data or task, such as leaf counting (Ubbens *et al*., 2018), pollen counting (Tello *et al*., 2018), or root architecture extraction (Yasrab *et al*., 2019).

Convolutional neural networks (CNNs) are a class of deep‐learning models that represent the state of the art for image analysis and are currently among the most popular methods in computer vision research. They are a type of multilayered neural network that uses convolution in at least one layer and are designed to process grid‐like data, such as images (LeCun *et al*., 2015). CNNs, such as U‐Net (Ronneberger *et al*., 2015), receive an image as input and then output another image, with each pixel in the output image representing a prediction for each pixel in the input image. CNNs excel at tasks such as segmentation and classification. They have been found to be effective for various tasks in agricultural research (Kamilaris & Prenafeta‐Boldú, 2018; Santos *et al*., 2020) and plant image analysis, including plant stress phenotyping (Jiang & Li, 2020), wheat spike counting (Pound *et al*., 2017), leaf counting (Ubbens & Stavness, 2017), and accession classification (Taghavi Namin *et al*., 2018).

For a CNN model to perform a particular task, it must be trained on a suitable dataset of examples. These examples are referred to as training data and are typically a collection of input images paired with the desired output for that image. In the case of segmentation, each input image is paired with a set of labels corresponding to each of the pixels in the input image. The process of creating such labelled training data is referred to as annotation, and this can be time consuming, as annotation of complex images can be labour intensive and many images may be desired, as larger training datasets typically result in improvements in trained model performance (Nakkiran *et al*., 2021).

Convolutional neural network model training involves a process called stochastic gradient descent (SGD) optimizing the parameters of a model such that the error is reduced. The error, commonly referred to as the loss, is a measure of the difference between the model's predictions and the correct labels for the training data examples. A separate validation dataset, consisting of similar examples, is used to provide information on the performance of the model during the training procedure on examples that have not been used as part of SGD optimization. Validation set performance may be used to decide when to stop training or assist in tuning hyperparameters, which are variables controlling fundamentals of the model that are not directly optimized by SGD.

Developing a CNN‐based system for a new image analysis task or dataset is challenging, because dataset design, model training, and hyperparameter tuning are time‐consuming tasks requiring competencies in both programming and machine learning.

Three questions that need answering when attempting a supervised learning project such as training a CNN are: how to split the data between training, validation, and test datasets; how to manually annotate or label the data; and how to decide how much data needs to be collected, labelled, and used for training in order to obtain a model with acceptable performance. The choices of optimal hyperparameters and network architecture are also considered to be a ‘black art’ requiring years of experience, and a need has been recognized to make the application of deep learning easier in practice (Smith, 2018).

The question of how much data to use in training and validation is explored in theoretical work that gives indications of a model's generalization performance based on dataset size and number of parameters (Vapnik, 2000). These theoretical insights may be useful for simpler models but provide an inadequate account of the behaviour of CNNs in practice (Zhang *et al*., 2017).

Manual annotation may be challenging, as proprietary tools may be used that are not freely available (Xu *et al*., 2019) and can increase the skill set required. Creating dense per‐pixel annotations for training is often a time‐consuming process. It has been argued that tens of thousands of images are required, making small‐scale plant‐image datasets unsuitable for training deep‐learning models (Ubbens *et al*., 2018).

The task of collecting datasets for the effective training of models is further confounded by the unique attributes of each dataset. All data are not created equal, with great variability in the utility of each annotated pixel for the model training process (Kellenberger *et al*., 2019). It may be necessary to add harder examples after observing weaknesses in an initial trained model (Soltaninejad *et al*., 2019), or to correct for a class imbalance in the data where many examples exist of a majority class (Buda *et al*., 2018).

Interactive segmentation methods using CNNs (e.g. Hu *et al*., 2018; Sakinis *et al*., 2019) provide ways to improve the annotation procedure by allowing user input to be used in the inference process and can be an effective way to create large high‐quality datasets in less time (Benenson *et al*., 2019).

When used in a semi‐automatic setting, such tools will speed up the labelling process but may still be unsuitable for situations where the speed and consistency of a fully automated solution are required. For example, when processing data from large‐scale root phenotyping facilities (e.g. Svane *et al*., 2019) where in the order of 100 000 images or more need to be analysed.

In this study we present and evaluate our software RootPainter, which makes the process of creating a dataset, training a neural network, and using it for plant image analysis accessible to ordinary computer users by facilitating all required operations with a cross‐platform, open‐source, and freely available user interface. The RootPainter software was initially developed for quantification of roots in images from rhizotron‐based root studies. However, we found its versatility to be much broader, with an ability to be trained to recognize many different types of structures in a set of images.

Although more root specific (Smith *et al*., 2020d; Gaggion *et al*., 2021; Narisetti *et al*., 2021) and more generalist segmentation tools such as Fiji (Schindelin *et al*., 2012) via DeepImageJ (Gómez‐de Mariscal *et al*., 2021) make it possible to run trained deep‐learning models for segmentation, they do not provide easy‐to‐use model training functionality, which is the purpose of the RootPainter software presented.

**Fig. 1:**
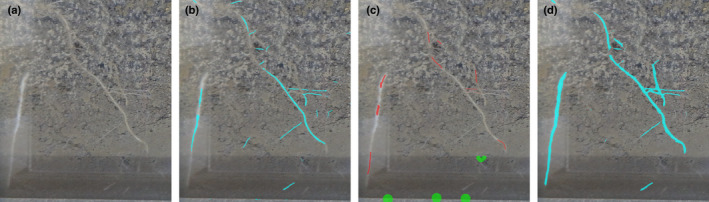
RootPainter corrective annotation concept. (a) Roots in soil. (b) Artificial intelligence (AI) root predictions (segmentation) shown in bright blue overlaid on photograph. (c) Human corrections of the initial segmentation, with corrections of false negatives shown in red and corrections of false positives shown in green. (d) After a period of training the AI learns from the corrections provided. The updated segmentation is shown in bright blue.

RootPainter allows a user to inspect model performance during the annotation process so they can make a more informed decision about how much and what data are necessary to label in order to train a model to an acceptable accuracy. It allows annotations to be targeted towards areas where the current model shows weakness (Fig. 1) in order to streamline the process of creating a dataset necessary to achieve a desired level of performance. RootPainter can operate in a semi‐automatic way, with a user assigning corrections to each segmented image, whilst the model learns from the assigned corrections, reducing the time‐requirements for each image as the process is continued. It can also operate in a fully automatic way by either using the model generated from the interactive procedure to process a larger dataset without required interaction, or in a more classical way by using a model trained from dense per‐pixel annotations which can also be created via the user interface.

We evaluate the effectiveness of RootPainter by training models for three different types of data and tasks without dataset‐specific programming or hyperparameter tuning. We evaluate the effectiveness on a set of rhizotron root images and, in order to evaluate the versatility of the system, on two other types of data, both involving objects in the images quite different from roots: a biopores dataset and a legume root nodules dataset.

For each dataset we compare the performance of models trained using the dense and corrective annotation strategies on images not used during the training procedure. If annotation is too time consuming, then RootPainter will be unfeasible for many projects. To investigate the possibility of rapid and convenient model training we use no prior knowledge and restrict annotation time to a maximum of 2 h for each model. We make two hypotheses. First, in a limited time period, RootPainter will be able to segment the objects of interest to an acceptable accuracy in three datasets including roots, biopores, and root nodules, demonstrated by a strong correlation between the measurements obtained from RootPainter and manual methods. Second, a corrective annotation strategy will result in a more accurate model compared with dense annotations, given the same time for annotation.

Training with corrective annotation is a type of interactive machine learning, as it uses a human in the loop in the model training procedure. As opposed to active learning, which involves the learner automatically selecting which examples the user labels (Settles, 2009), interactive machine learning involves a human deciding which examples should be added for future iterations of training (Amershi *et al*., 2014).

Prior work for interactive training for segmentation includes Gonda *et al*. (2017) and Kontogianni *et al*. (2019). Gonda *et al*. (2017) evaluated their method using neuronal structures captured using electron microscopy and found the interactively trained model produced better segmentations than a model trained using exhaustive ground‐truth labels.

Kontogianni *et al*. (2019) combined interactive segmentation with interactive training by using the user feedback in model updates. Their training approach requires an initial dataset with full ground‐truth segmentations, whereas our method requires no prior labelled data, which was a design choice we made to increase the applicability of our method to plant researchers looking to quantify new objects in a captured image dataset.

As opposed to Gonda *et al*. (2017) we use a more modern, fully convolutional network model, which we expect to provide substantial efficiency benefits when dealing with larger images. Our work is novel, in that we evaluate an interactive corrective annotation procedure in terms of annotation time to reach a certain accuracy on real‐world plant‐image datasets. Synthetic data are often used to evaluate interactive segmentation methods (Benard & Gygli, 2017; Li *et al*., 2018; Mahadevan *et al*., 2018). To provide more realistic measurements of annotation time we use real human annotators for our experiments. As opposed to many competing deep‐learning methods for segmentation, we provide a graphical user interface that allows all operations to be completed using a user interface, an essential feature for ensuring uptake in the plant image analysis community.

### Roots in soil

Plant roots are responsible for uptake of water and nutrients. This makes understanding root system development critical for the development of resource‐efficient crop production systems. For this purpose, we need to study roots under real‐life conditions in the field, studying the effects of crop genotypes and their management (Rasmussen *et al*., 2015; Rasmussen & Thorup‐Kristensen, 2016), cover crops (Thorup‐Kristensen, 2001), crop rotation (Thorup‐Kristensen *et al*., 2012), and other factors. We need to study deep rooting, as this is critical for the use of agriculturally important resources, such as water and nitrogen (N) (Thorup‐Kristensen & Kirkegaard, 2016; Thorup‐Kristensen *et al*., 2020a).

Rhizotron‐based root research is an important example of plant research. Acquisition of root images from rhizotrons is widely adopted (Rewald *et al*., 2012), as it allows repeated and nondestructive quantification of root growth and often to the full depth of the root systems. Traditionally, the method for root quantification in such studies involves a lengthy procedure to determine the root density on acquired images by counting intersections with grid lines (Thorup‐Kristensen, 2006).

Manual methods require substantial resources and can introduce undesired inter‐annotator variation on root density; therefore, a faster and more consistent method is required. More recently, fully automatic approaches using CNNs have been proposed (Smith *et al*., 2020d); although effective, such methods may be challenging to repurpose to different datasets for root scientists without the required programming expertise. A method that made the retraining process more accessible and convenient would accelerate the adoption of CNNs within the root research community.

### Biopores

Biopores are tubular or round‐shaped continuous voids formed by root penetration and earthworm movement (Kautz, 2015). They function as preferential pathways for root growth (Han *et al*., 2015b) and are therefore important for plant resource acquisition (Kopke *et al*., 2015; Han *et al*., 2017). Investigation of soil biopores is often done by manually drawing on transparent sheets on an excavated soil surface (Han *et al*., 2015a). This manual approach is time consuming and precludes a more in‐depth analysis of detailed information, including diameter, surface area, or distribution patterns such as clustering.

### Root nodules

Growing legumes with N‐fixing capacity reduces the use of fertilizer (Kessel *et al*., 2000); hence, there is an increased demand for legume‐involved intercropping (Hauggaard‐Nielsen *et al*., 2001) and precropping for carryover effects. Roots of legumes form associations with rhizobia, forming nodules on the roots, where the N fixation occurs. Understanding the nodulation process is important to understand this symbiosis and the N fixation. However, counting nodules from the excavated roots is a cumbersome and time‐consuming procedure, especially for species with many small nodules, such as clovers (*Trifolium* spp.).

## Materials and Methods

### Software implementation

RootPainter uses a client–server architecture, allowing users with a typical laptop or desktop computer to utilize a graphics processing unit (GPU) on a more computationally powerful server. The client and server can be used on the same machine if it is equipped with suitable hardware, reducing network input/output overhead. Instructions are sent from the client to server using human‐readable JSON (JavaScript Object Notation) format. The client–server communication is facilitated entirely with files via a network drive or file synchronization application. This allows utilization of existing authentication, authorization and backup mechanisms whilst removing the need to setup a publicly accessible static Internet Protocol address. The graphical client is implemented using PyQt5 which binds to the Qt cross‐platform widget toolkit. The client installers for MacOS, Windows, and Linux are built using the PyInstaller build system, which bundles all required dependencies. As opposed to the more generalist annotation software napari (Sofroniew *et al*., 2022), which is also built using Qt, the RootPainter client is designed to specifically facilitate our proposed corrective annotation protocol and to not require Python familiarity. Image data can be provided as JPEG, PNG, or TIF and in either colour or greyscale. Image annotations and segmentations are stored as PNG files. Models produced during the training process are stored in the Python pickle format and extracted measurements in comma‐separated value (CSV) text files.

A folder referred to as the ‘sync directory’ is used to store all datasets, projects, and instructions that are shared between the server and client. The server setup (Supporting Information Notes S1) requires familiarity with the Linux command line, so should be completed by a system administrator. The server setup involves specification of a sync directory, which must then be shared with users. Users will be prompted to input the sync directory relative to their own file system when they open the client application for the first time and it will be automatically stored in their home folder in a file named *root_painter_settings.json*, which the user may delete or modify if required.

#### Creating a dataset

The ‘Create training dataset’ functionality is available as an option when opening the RootPainter client application. It is possible to specify a source image directory, which may be anywhere on the local file system and whether all images from the source directory should be used or a random sample of a specified number of images. It is also possible to specify the target width and height of one or more samples to take from each randomly selected image; this can provide two advantages in terms of training performance. First, RootPainter loads images from disk many times during training, which for larger images (> 2000 × 2000 px^2^) can slow down training in proportion to image size and hardware capabilities. Second, recent results (Lin *et al*., 2020) indicate that capturing pixels from many images is more useful than capturing more pixels from each image when training models for semantic segmentation; thus, when working with datasets containing many large images, using only a part of each image will likely improve performance given a restricted time for annotation.

When generating a dataset, each image to be processed is evaluated for whether it should be split into smaller pieces. If an image's dimensions are close to the target width and height, then the image will be added to the dataset without it being split. If an image is substantially bigger, then all possible ways to split the image into equally sized pieces above the minimum are evaluated. For each of the possible splits, the resultant piece dimensions are evaluated in terms of their ratio distance from a square and distance from the target width and height. The split that results in the smallest sum of these two distances is then applied. From the split image, up to the *maximum tiles per image* are selected at random and saved to the training dataset. The source images do not need to be the same size, and the images in the generated dataset will not necessarily be the same size, but all images provided must have a width and height of at least 572 px; we recommend at least 600 px, as this will allow random crop data augmentation. The dataset is created in the RootPainter sync directory in the datasets folder in a subdirectory that takes the user‐specified dataset name. To segment images in the original dimensions, the dataset creation routine can be bypassed by simply copying or moving a directory of images into a subdirectory in the RootPainter datasets directory.

#### Working with projects

Projects connect datasets with models, annotations, segmentations, and messages returned from the server. They are defined by a project file (.seg_proj), which specifies the details in JSON, and a project folder containing relevant data. The options to create a project or open an existing project are presented when opening the RootPainter client application. Creating projects requires specifying a dataset and, optionally, an initial model file. Alternatively, a user may select ‘random weights’, also known as training from scratch, which will use He initialization (He *et al*., 2015b) to assign a model's initial weights. A project can be used to inspect the performance of a model on a given dataset in the client or to train a model with new annotations, which can also be created using drawing tools in the client user interface.

#### Model architecture

We modified the network architecture from Smith *et al*. (2020d), which is a variant of U‐Net (Ronneberger *et al*., 2015) implemented in PyTorch (Paszke *et al*., 2017) using group normalization (Wu & He, 2018) layers. U‐Net is composed of a series of down‐blocks and up‐blocks joined by skip connections. The entire network learns a function that converts the input data into a desired output representation; for example, from an image of soil to a segmentation or binary map indicating which of the pixels in the image are part of a biopore. In the down‐blocks we added 1×1 convolution to halve the size of the feature maps. We modified both down‐blocks and up‐blocks to learn residual mappings, which have been found to ease optimization and improve accuracy in CNNs (He *et al*., 2015a) including U‐Net (Zhang *et al*., 2018). To speed up inference by increasing the size of the output segmentation, we added 1 px padding to the convolutions in the down‐blocks and modified the input dimensions from 512×512×3 to 572×572×3, which resulted in a new respective output size of 500×500×2, containing a channel for the foreground and background predictions. The modified architecture has *c*. 1.3 million trainable parameters, whereas the original had 31 million. These alterations reduced the saved model size from 124.2 MB (Smith *et al*., 2019b) to 5.3 MB, making it small enough to be conveniently shared via email.

#### Creating annotations

Annotations can be added by drawing in the user interface with either the foreground or background brush tools. It is also possible to undo or redo brush strokes. Annotation can be removed with the eraser tool. If an image is only partially annotated, then only the regions with annotation assigned will be used in the training. Whilst annotating, it is possible to hide and show the annotation, image or segmentation. For convenience during use, a table of keyboard shortcuts is presented in Notes S2. When the user clicks ‘Save & next’ in the interface, the current annotation will be saved and synced with the server, ready for use in training. The first and second annotations are added to the training and validation sets respectively (see the Training procedure section). Afterwards, to maintain a typical ratio between training and validation sets, annotations will be added to the validation set when the training set is at least five times the size of the validation set, otherwise they will be added to the training set.

#### Training procedure

The training procedure can be started by selecting ‘Start training’ from the network menu, which will send a JSON instruction to the server to start training for the current project. The training will only start if the project has at least two saved annotations, as at least one is required for each of the training and validation sets. Based on Smith *et al*. (2020d) we use a learning rate of 0.01 and Nestorov momentum with a value of 0.99. We removed weight decay, as results have shown similar performance can be achieved with augmentation alone whilst reducing the coupling between hyperparameters and dataset (Hernández‐García & König, 2019). The removal of weight decay has also been suggested in practical advice (Bengio, 2012) based on earlier results (Collobert & Bengio, 2004) indicating its superfluity when early stopping is used. We do not use a learning rate schedule in order to facilitate an indefinitely expanding dataset.

An *epoch* typically refers to a training iteration over the entire dataset (Goodfellow *et al*., 2016). In this context, we initially define an epoch to be a training iteration over 612 image subregions corresponding to the network input size. The images to be used in the network training procedure are sampled randomly with replacement from all training set images. We found an iteration over this initial epoch size to take *c*. 30 s using two RTX 2080 Ti GPUs with an automatically selected batch size of 6. If the training dataset expands beyond 306 images, then the number of sampled subregions per epoch is set to twice the number of training images, to avoid validation overwhelming training time. The batch size is automatically selected based on total GPU memory, and all GPUs will be used by default using data parallelism.

RootPainter utilizes a supervised learning procedure, which involves training a model to predict the same value (either foreground or background) for a pixel in the input data as the one found in the corresponding location of an annotation. The annotations are created by the user and include pixels annotated as foreground, pixels annotated as background, and pixels without annotation. Only the pixels annotated as foreground or background are used in training. This is achieved by setting the pixels without foreground or background annotation to 0 in both the network prediction and associated annotation. The distance between the annotated pixels and network predictions is computed using a loss function, which is a combination of dice‐loss and cross‐entropy taken from Smith *et al*. (2020d). This computed loss is then used to update the network weights, leading to a model with reduced error on subsequent images.

After each epoch, the model predictions are computed on the validation set and *F*_1_ is calculated for the current and previously saved model. If the current model's *F*_1_ is higher than the previously saved model, then it is saved with its number and current time in the file name. If, for 60 epochs, no model improvements are observed and no annotations are saved or updated, then training will stop automatically.

We designed the training procedure to have minimal RAM requirements that do not increase with dataset size, in order to facilitate training on larger datasets. We found the server application to use < 8 GB of RAM during training and inference and would suggest at least 16 GB RAM for the machine running the server application. We found the client to use < 1 GB RAM but have not yet tested on devices equipped with < 8 GB of RAM.

#### Augmentation

We modified the augmentation procedure from Smith *et al*. (2020d) in three ways. We changed the order of the transforms from fixed to random in order to increase variation. We reduced the probability that each transform is applied to 80% in order to reduce the gap between clean and augmented data, which recent results indicate can decrease generalization performance (He *et al*., 2019). We also modified the elastic grid augmentation, as we found the creation of the deformation maps to be a performance bottleneck. To eliminate this bottleneck we created the deformation maps at an eighth of the image size and then interpolated them up to the correct size.

#### Creating segmentations

It is possible to view segmentations for each individual image in a dataset by creating an associated project and specifying a suitable model. The segmentations are generated automatically via an instruction sent to the server when viewing each image and saved in the segmentations folder in the corresponding project.

When the server generates a segmentation, it first segments the original image and then a horizontally flipped version. The output segmentation is computed by taking the average of both and then thresholding at 0.5. This technique is a type of test time data augmentation, which is known to improve performance (Perez *et al*., 2018). The segmentation procedure involves first splitting the images into tiles with a width and height of 572 px, which are each passed through the network, and then an output corresponding to the original image is reconstructed.

It is possible to segment a larger folder of images using the ‘Segment folder’ option available in the network menu. To do this, an input directory, output directory, and one or more models must be specified. The model with the highest number for any given project will have the highest accuracy in terms of *F*_1_ on the automatically selected validation set. Selecting more than one model will result in model averaging, an ensemble method that improves accuracy as different models do not usually make identical errors (Goodfellow *et al*., 2016). Selecting models from different projects representing different training runs on the same dataset will likely lead to a more diverse, and thus more accurate, ensemble, given they are of similar accuracy. It is also possible to use models saved at various points from a single training run, a method that can provide accuracy improvements without extending training time (Sennrich *et al*., 2016).

**Fig. 2:**
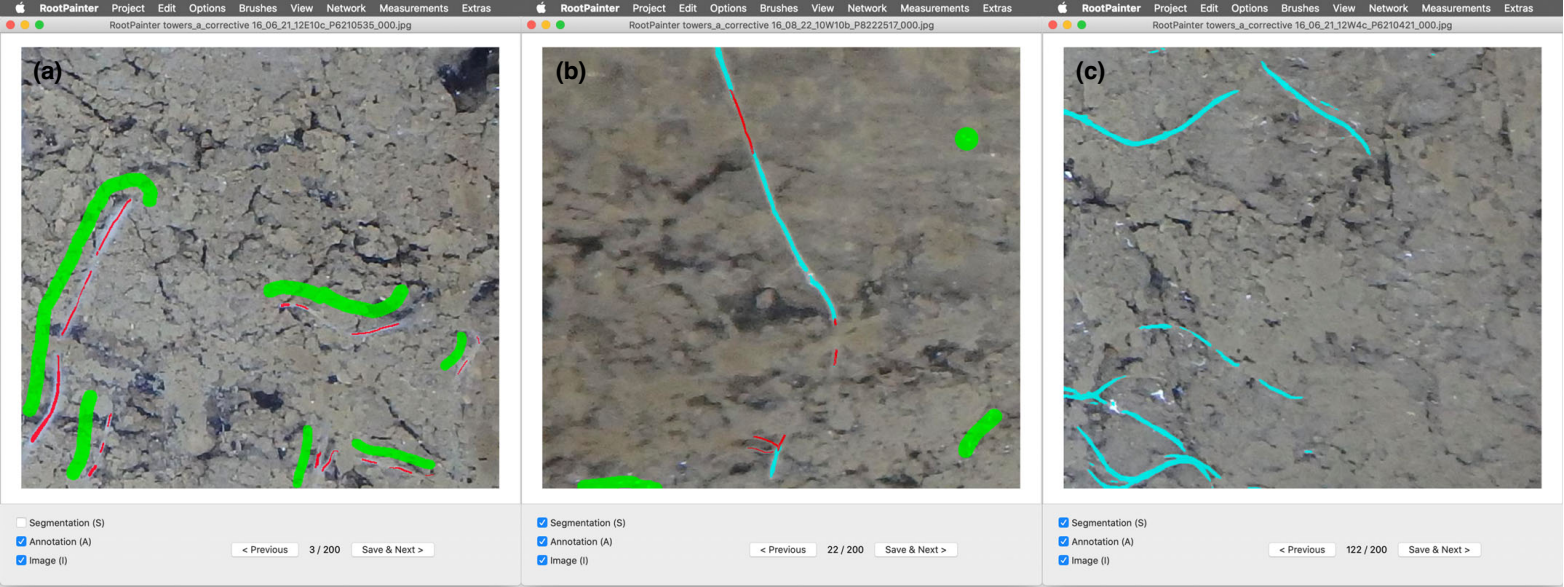
Screenshots of the RootPainter software, showing examples of the various stages of the interactive training procedure. (a) Initial annotation created at the start of interactive training, with foreground annotation shown in red and background annotation shown in green. (b) Corrective annotation whilst the model progresses to a suitable solution. The segmentation is shown in light blue. (c) Segmentation shown later in the model training process.

Fig. 2 shows the user interface with various stages of the interactive training procedure.

#### Extracting measurements

It is possible to extract measurements from the segmentations produced by selecting an option from the measurements menu. The ‘Extract length’ option extracts centrelines using the skeletonize method from scikit‐image (Walt *et al*., 2014) and then counts the centreline pixels for each image. The ‘Extract region properties’ uses the scikit‐image regionprops method to extract the coordinates, diameter, area, perimeter, and eccentricity for each detected region and stores this along with the associated filename. The ‘Extract count’ method gives the count of all regions per image. Each of the options requires the specification of an input segmentation folder and an output CSV.

### Datasets

#### Biopore images

Biopore images were collected near Meckenheim (50°37′9″N, 6°59′29″E) at a field trial of the University of Bonn in 2012; see Han *et al*. (2015a) for a detailed description. Within each plot, an area was excavated to a depth of 0.45 m. The exposed soil surface was carefully flattened to reveal biopores and then photographed in colour.

Bersoft software (v.7.25; Windows) was used for biopore quantification. Using the eclipse function, the visible biopores were marked, and then the count number was generated as a CSV file. Pores < 2 mm were excluded from biopore counting.

We restricted the analysis to images with a suitable resolution and cropped to omit border areas. For each image, the number of pixels per millimetre was recorded using Gimp (v.2.10, MacOS) in order to calculate pore diameter. We split the images into two folders: BP_counted, which contained 39 images and was used for model validation after training, as these images had been counted by a biopore expert, and BP_uncounted, which contained 54 images and was used for training.

#### Nodule images

Root images of Persian clover (*Trifolium resupinatum*) were acquired at 800 DPI in colour using a water‐bed scanner after root extraction. We used a total of 113 images that all had a blue background but were taken with two different scanners. From the 113 images, 65 were captured using an Epson V700 scanner (Epson, Nagano, Japan) and appeared darker and underexposed, whereas 48 were captured using an Epson Expression 12000XL Photo Scanner and appeared well lit, showing the nodules more clearly. The blue background was obtained by blocking the scanner transparency unit with nontransparent blue paper to create a background that provided contrast to the root nodules. Blocking the scanner transparency unit meant that the only light source was from the document table (the lower part) of the scanner.

They were counted manually using WinRhizo Pro (v.2016; Regent Instruments Inc., Sainte‐Foy, QC, Canada). Image sections were enlarged, and nodules were selected manually by clicking. Then, the total number of marked nodules were counted by the software. We manually cropped to remove the borders of the scanner using Preview (v.10.0; MacOS) and converted to JPEG to ease storage and sharing. Of these, 50 were selected at random to have subregions included in training, and the remaining 63 were used for validation.

#### Roots dataset

We downloaded the 867 grid‐counted images and manual root length measurements from Smith *et al*. (2019a), which were made available as part of the evaluation of U‐Net for segmenting roots in soil (Smith *et al*., 2020d) and originally captured as part of a study on chicory drought stress (Rasmussen *et al*., 2020) using a 4 m rhizobox laboratory described in Thorup‐Kristensen *et al*. (2020b). We removed the 10 test images from the grid‐counted images, leaving 857 images. The manual root length measurements are a root intensity measurement per image, which was obtained by counting root intersections with a grid as part of Rasmussen *et al*. (2020).

### Annotation and training

For the roots, nodules, and biopores, we created training datasets using the ‘Create training dataset’ option. We used a random sample, with the details specified in Table 1. The two users (user a and user b) that we used to test the software were the two first authors. Each user trained two models for each dataset. For each model, the user had 2 h (with a 30 min break between them) to annotate 200 images. We first trained a model using the corrective annotation strategy whilst recording the finish time; we then repeated the process with the dense annotation strategy, using the recorded time from the corrective training as a time limit. This was done to ensure the same annotation time was used for both annotation strategies. With corrective annotations, the annotation and training processes are coupled, as there is a feedback loop between the user and model being trained that happens in real time, whereas with dense annotation the user annotated continuously, without regard to model performance. The protocol followed when using corrective annotations is outlined in Notes S3, and annotation advice given in Notes S4. For the first six annotations on each dataset, we added clear examples rather than corrections. This was because we observed divergence in the training process when using corrective annotation from the start in preliminary experiments. We suspect the divergence was caused by the user adding too many background classes compared with foreground or difficult examples. When creating dense annotations, we followed the procedure described in Notes S5.

**Table 1:** Details for each of the datasets created for training.

Object	Name	Source folder	Reference	To sample	Max. tiles	Target size
Biopores	BP_750_training	BP_uncounted	Smith *et al*. (2020b)	50	4	750
Nodules	nodules_750_training	counted_nodules	Smith *et al*. (2020a)	50	4	750
Roots	towers_750_training	grid_counted_roots	Smith *et al*. (2019a)	200	1	750

The numbers of images and tiles were chosen to enable a consistent dataset size of 200 images. Only 50 images were sampled for the biopores and nodules, in order to ensure there were enough images left in the test set. The datasets created are available to download from Smith *et al*. (2020c).

**Fig. 3:**
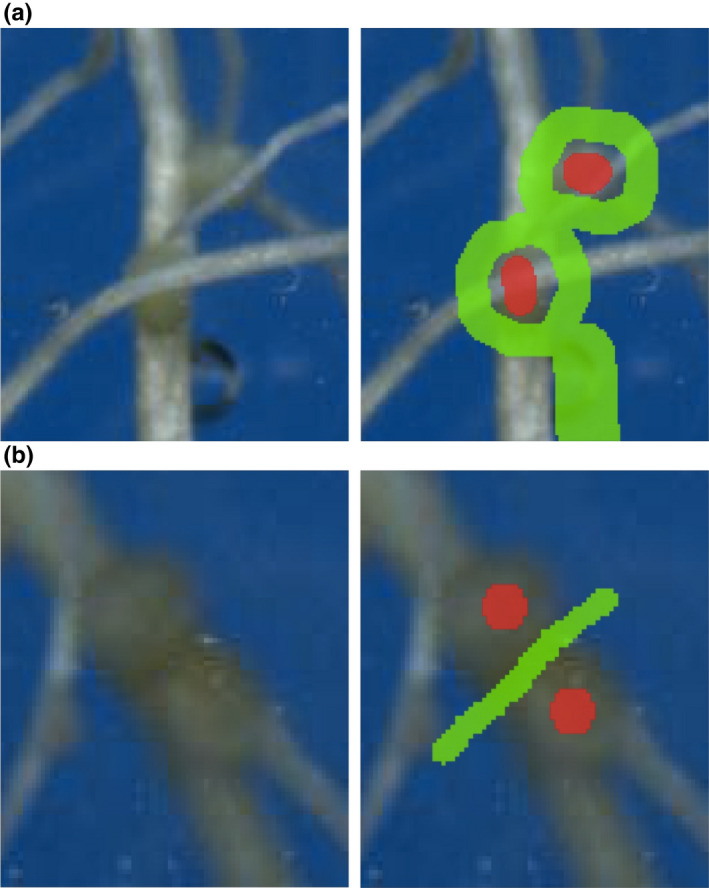
Nodule annotation. The red brush was used to mark the foreground (nodules) and the green brush to mark the background (not nodules). (a) We annotated nodules occluded by roots as though the roots were not there. (b) Adjacent nodules were separated using the background class.

When annotating roots, in the interests of efficiency, a small amount of soil covering the root would still be considered as root if it was very clear that root was still beneath. Larger gaps were not labelled as root. Occluded parts of nodules were still labelled as foreground (Fig. 3a). Only the centre part of a nodule was annotated, leaving the edge as undefined. This was to avoid nodules that were close together being joined into a single nodule. When annotating nodules that were touching, a green line (background labels) was drawn along the boundary to teach the network to separate them so that the segmentation would give the correct counts (Fig. 3b).

After completing the annotation, we left the models to finish training using the early stopping procedure and then used the final model to segment the respective datasets and produce the appropriate measurements.

We also repeated this procedure for the projects, but using a restricted number of annotations by limiting to those that had been created in just 30, 60, 90, 120, and 150 min (including the 30 min break period) to give us an indication of model progression over time with the two different annotation strategies.

### Measurement, correlation, and segmentation metrics

For each project we obtained correlations with manual measurements using the portion of the data not used during training to give a measure of generalization error, which is the expected value of the error on new input (Goodfellow *et al*., 2016). For the roots dataset, the manual measurements were compared with length estimates given by RootPainter, which are obtained from the segmentations using skeletonization and then pixel counting.

For the biopores and nodules datasets we used the extract region properties functionality from RootPainter, which gives information on each connected region in an output segmentation. For the biopores, the regions < 2 mm in diameter were excluded. The numbers of connected regions for each image were then compared with the manual counts.

In order to obtain segmentation metrics, we used the extract segmentation metrics function available from the RootPainter extras menu. This function generates a CSV file containing dice score, recall, precision, and accuracy for each of the segmented images in a project. The ground truth used for evaluation is the model prediction with the corrections assigned (i.e. the corrected segmentation). This corrected segmentation is then used to evaluate the predicted segmentation, which is stored in the segmentations folder.

### Filtering nodules by size

We investigated the effect of filtering out nodules less than a certain size by computing the correlation between the automated and manual nodule counts as a function of a size threshold. The size threshold meant that counted nodules would include only those above a specific area in pixels. We computed the correlation with each size threshold from 0 to 400 px. We did this for the model trained by user b only.

## Results

We report the *R* ^2^ for each annotation strategy for each user and dataset (Table 2). Training with corrective annotations resulted in strong correlation ($R^{2}\geq 0.7$) between the automated measurements and manual measurements five out of six times. The exception was the nodules dataset for user b with an *R* ^2^ of 0.69 (Table 2). Training with dense annotations resulted in strong correlation three out of six times, with the lowest *R* ^2^ being 0.55 also given by the nodules dataset for user b (Table 2).

**Table 2:** *R* ^2^ for each training run.

Dataset	User	Corrective *R* ^2^	Dense *R* ^2^
Biopores	a	0.78	0.58
Biopores	b	0.78	0.67
Nodules	a	0.73	0.89
Nodules	b	0.69	0.55
Roots	a	0.89	0.90
Roots	b	0.92	0.90

These are computed by obtaining measurements from the segmentations from the final trained model and then correlating with manual measurements for the associated dataset.

**Fig. 4:**
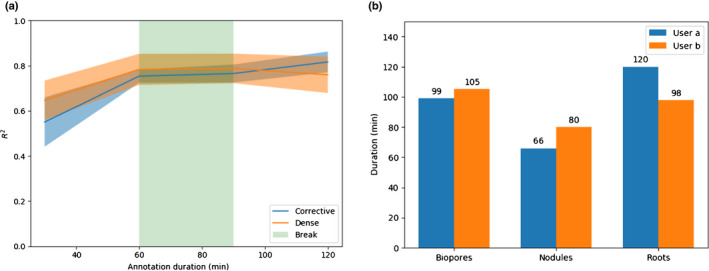
Annotation duration and accuracy. (a) Mean and SE for the *R* ^2^ values over time. These include the 30 min break and are restricted to time points where multiple observations are available. The shaded area indicates the SE. (b) User‐reported duration in minutes for annotating each dataset, excluding the 30 min break taken after 1 h of annotation. The annotator would use the same amount of time for both corrective and dense annotation strategies. The time fell below the limit of 2 h (excluding break) when they ran out of images to annotate.

For each annotation strategy, we report both the mean and SE for the *R* ^2^ values obtained from all datasets and both users (Table 3). The mean of the *R* ^2^ values obtained when using corrective annotation shows they tended to be higher than with dense annotation, but the differences were not statistically significant (mixed‐effects model; *P* ≤ 0.05). We plot the mean and SE at each time point for which multiple *R* ^2^ values were obtained (Fig. 4a). In general corrective improved over time, overtaking dense performance just after the break in annotation (Fig. 4a). The 30 min break period taken by the annotator after 1 h corresponds to a flat line in performance during that period (Fig. 4a). On average, dense annotations were more effective at the 30 min time period, whereas corrective annotations were more effective after 2 h (including the 30 min break) and at the end of the training (Table 3).

**Table 3:** Mean and SE of the *R* ^2^ for each annotation strategy.

Strategy	Mean	SE
Corrective	0.80	0.04
Dense	0.75	0.07

These are computed by obtaining measurements from the segmentations from the final trained model and then correlating with manual measurements. Using a mixed‐effects model with annotation strategy as a fixed factor and user and dataset as random factors, no significant effects were found (P≤0.05).

We report the duration for each user and dataset (Fig. 4b). Five out of six times all 200 images were annotated in less than the 2 h time limit. The nodules dataset took the least time, with annotation completed in 66 min and 80 min for users a and b, respectively (Fig. 4b). The roots dataset for user a was the only project where the 2 h time limit was reached without running out of images (Fig. 4b).

**Fig. 5:**
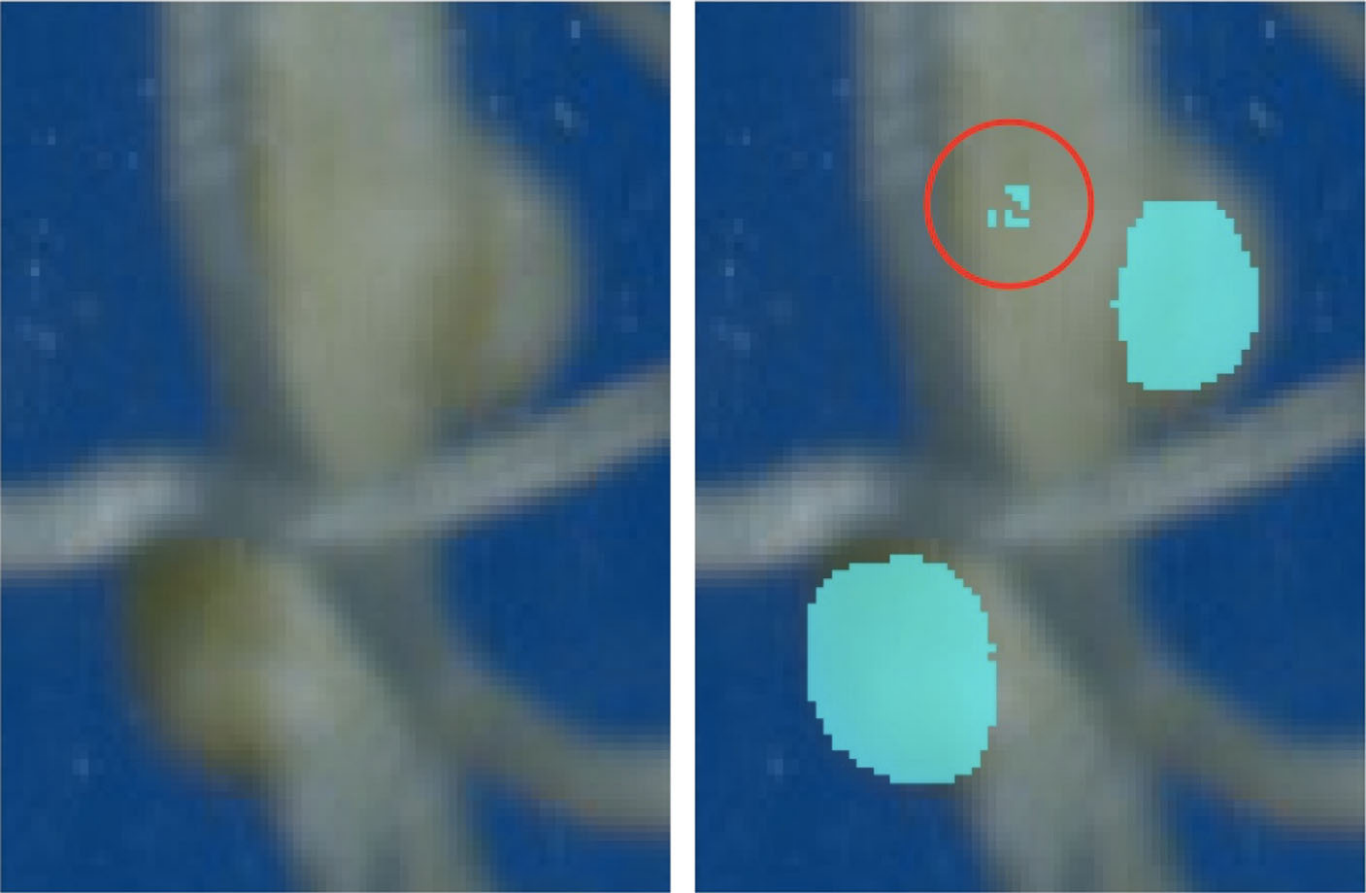
Two correctly detected nodules shown with three false positives. Segmentation is shown overlaid in light blue on top of a subregion of one of the nodule images used for evaluation. The correct nodules are much larger and on the edge of the root. The three false positives are indicated by a red circle. They are much smaller and bunched together.

We show an example of errors found from the only model trained correctively that did not result in a strong correlation (Fig. 5). There were cases when the vast majority of pixels were labelled correctly, but a few small incorrect pixels could lead to substantial errors in count (Fig. 5).

We found filtering nodules less than a certain size to provide substantial reductions in error. There was an improvement in *R* ^2^ from 0.69 to 0.75 when changing the area threshold from 0 to 5 px. The benefits increased up to an area threshold of 284 px, giving an *R* ^2^ of 0.9 (Notes S6).

**Fig. 6:**
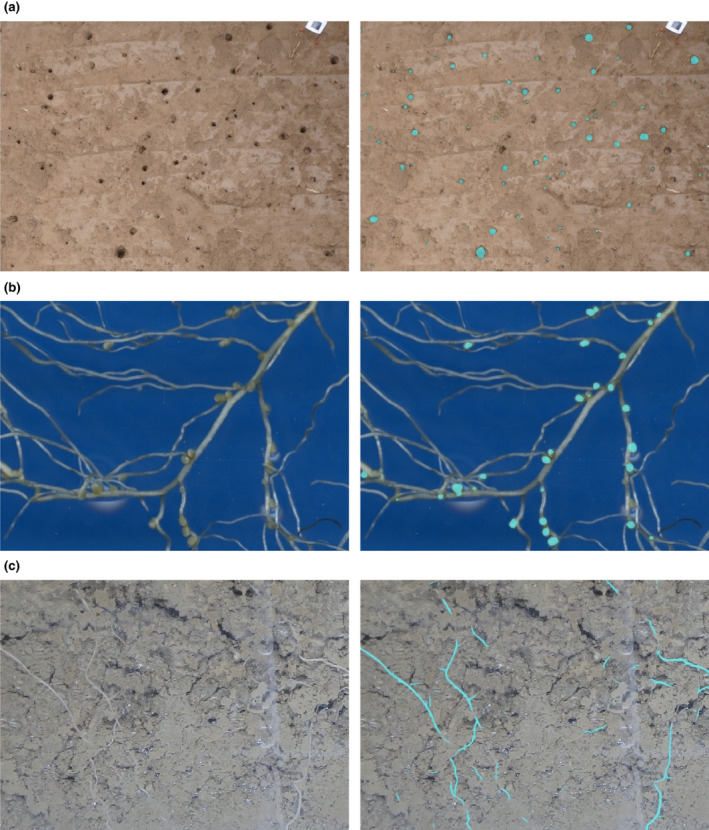
Example input and segmentation output from photographs not used in training. The segmentations are shown in light blue and are from models trained from scratch using no prior knowledge with annotations created using the corrective annotation protocol with RootPainter. (a) Biopores. Annotations created by user b in 1 h 45 min. (b) Nodules. Annotations created by user a in 1 h 6 min. (c) Roots. Annotations created by user a in 2 h.

**Fig. 7:**
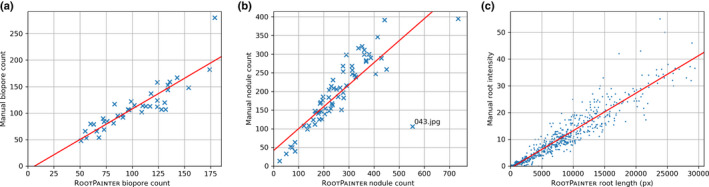
Manual measurements plotted against automatic measurements attained using RootPainter. (a) Biopores using user b corrective model. (b) Nodules using user a corrective model. (c) Roots in soil using user a corrective model.

We show examples of accurate segmentation results obtained with models trained using the corrective annotation strategy (Fig. 6a–c) along with the corresponding manual measurements plotted against the automatic measurements obtained using RootPainter (Fig. 7).

The observed *R* ^2^ values for corrective annotation had a significant positive correlation with annotation duration (*P* < 0.001). There was no significant correlation between annotation time and *R* ^2^ values for models trained using dense annotations.

**Fig. 8:**
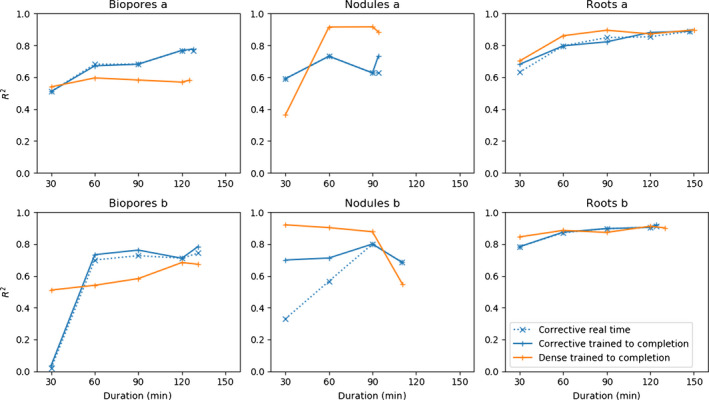
*R* ^2^ for the annotations attained after 30, 60, 90, and 120 min and the final time point for users a and b on the three datasets for dense and corrective annotation strategies. ‘Trained to completion’ refers to models that were trained until stopping without interaction, using the annotations created within the specified time period, whereas ‘real time’ refers to models saved during the corrective annotation procedure as it happened. For the corrective annotations we plot both the performance of the model saved during the training procedure and the same model if allowed to train to completion with the annotations available at that time.

We plot the *R* ^2^ for each project after training was completed along with the *R* ^2^ obtained with training done only on annotations at restricted time limits, and refer to these as *trained to completion*, along with the models saved at that time point during the corrective annotation procedure as it happened, which we refer to as *real time* (Fig. 8). After only 60 min of annotation, all models trained for roots in soil gave a strong correlation with grid counts (Fig. 8, roots a and b). The performance of dense annotation for user b on the nodules dataset was anomalous, with a decrease in *R* ^2^ as more annotated data were used in training (Fig. 8, nodules b). The corrective models obtained in real time were similar to those trained to completion, except nodules by user b, indicating that computing power was sufficient for real‐time corrective training (Fig. 8).

**Fig. 9:**
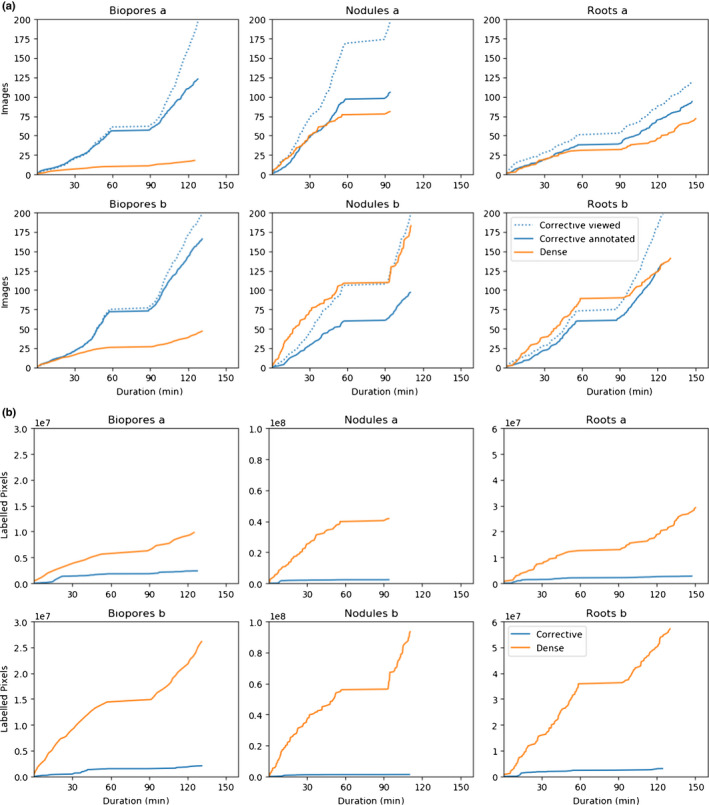
Annotation progression. (a) Number of images viewed and annotated for the dense and corrective annotation strategies. For dense annotation, all images are both viewed and annotated, whereas corrective annotations are only added for images where the model predictions contain clear errors. (b) Total number of annotated pixels for dense and corrective annotation strategies over time during the annotation procedure. For dense annotation, almost all pixels in each image are annotated. Corrective annotations are only applied to areas of the image where the model being trained exhibits errors.

We plot the number of images viewed and annotated for the corrective and dense annotation strategies (Fig. 9a). For the corrective annotation strategy, only some of the viewed images required annotation. In all cases the annotator was able to progress through more images using corrective annotation (Fig. 9a).

For the roots and nodules datasets for user b for the first hour of training, progress through the images was faster when performing dense annotation (Fig. 9a, roots b and nodules b).

**Fig. 10:**
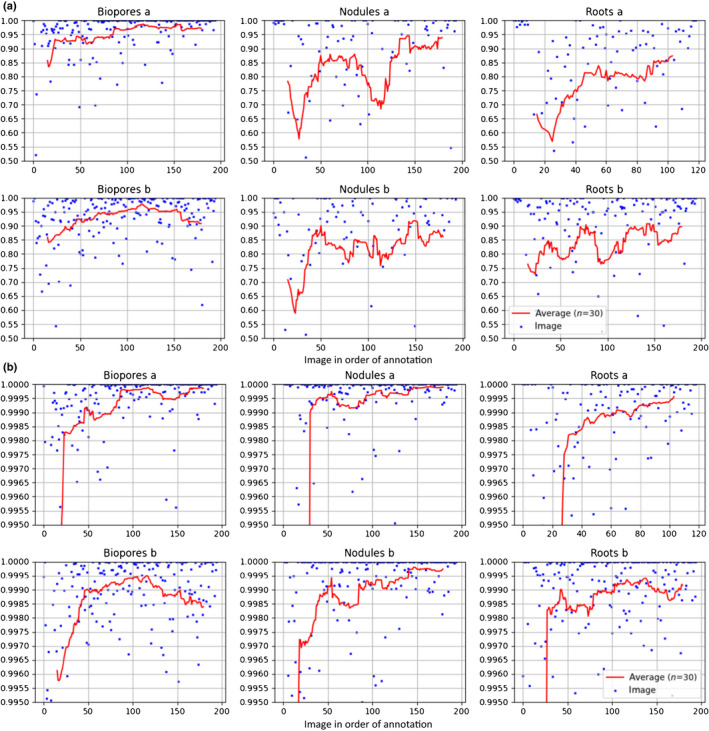
Segmentation metrics for each of the images segmented as part of the interactive segmentation procedure in order of annotation: (a) dice score; (b) accuracy. Metrics were computed using the segmentations and corrective annotations created as part of the interactive training procedure for user a and user b for each of the three datasets. In this case the ground truth used for evaluation is the model prediction with the corrections assigned (i.e. the corrected segmentation). Dice score ranges from 0 to 1 and is higher when the model prediction agrees with the ground truth. Accuracy ranges from 0 to 1 and is the ratio of pixels that were predicted correctly to the total pixels in the image. As accuracy is very high, to show the changes in the moving average, accuracy is only shown in the range of 0.99 to 1.0. There is a trend of continuous improvement as more images are annotated and interactive training continues.

We plot the number of labelled pixels for each training procedure over time for both corrective and dense annotations (Fig. 9b). With corrective annotation, fewer pixels were labelled in the same time period, and as the annotator progressed through the images the rate of label addition decreased (Fig. 9b). The dice score (Fig. 10a) and accuracy (Fig. 10b) are also plotted, along with running averages (*n* = 30) for the models trained using corrective annotation. For both dice and accuracy, the running average shows both large fluctuations and a trend of continuous improvement as more images are annotated. The performance of the biopores model being trained by user b is an outlier, as it appears to decrease in accuracy and to a lesser extent dice score towards the end of training. For the nodules and roots datasets, towards the end of corrective annotation, the dice score is approximately 0.9 but with larger fluctuations, whereas for the biopores the dice score appears to stay consistently above 0.9.

## Discussion

In this study, we focused on annotation duration, as we consider the time requirements for annotation rather than the number of available images to be more relevant to the concerns of the majority of plant research groups looking to use deep learning for image analysis. Our results, for corrective training in particular, confirm our first hypothesis by showing that a deep‐learning model can be trained to a high accuracy for the three different datasets of varying target objects, background, and image quality in < 2 h of annotation time.

Our results demonstrate the feasibility of training an accurate model using annotations made in a short time period, which challenges the claims that tens of thousands of images (Ubbens *et al*., 2018) or substantial labelled data (Narisetti *et al*., 2019) are required to use CNNs. In practice, we also expect longer annotation periods to provide further improvement. The *R* ^2^ for corrective training had a significant correlation with annotation duration, indicating that spending more time annotating would continue to improve performance.

There was a trend for an increasing fraction of viewed images to be accepted without further annotation later in the corrective training (Fig. 9a), indicating fewer of the images required corrections as the model performance improved. This aligns with the reduction in the rate of growth for the total amount of corrections (Fig. 9b), indicating continuous improvement in the model accuracy over time during the corrective training.

We suspect the cases where dense annotation had a comparatively faster speed in the beginning (Fig. 9a, roots b and nodules b) were due to three factors. First, switching through images has little overhead when using the dense annotation strategy as there is no delay caused by waiting for segmentations to be returned from the server. Second, corrective annotation will take a similar amount of time to dense annotation in the beginning as the annotator needs to assign a large amount of corrections for each image. And third, many of the nodule images did not contain nodules, meaning dense annotations could be added almost instantly.

The average dice scores of approximately 0.9 for roots (Fig. 10a) are similar to previous results for root segmentation (Smith *et al*., 2020d), indicating our trained model was accurate (please see later note on the limitations of such comparisons). For comparison, in previous work, similar root segmentation accuracy was obtained using approximately 25 h of root annotation time (Smith *et al*., 2020d). We found that the dice scores for biopores were higher than those for roots or nodules (Fig. 10a), which we suspect was due to the biopore dataset being easier to segment, with higher contrast between the biopore and surrounding soil. Our reported dice‐scores plots (Fig. 10a) serve as a point of comparison for future work, and in combination with RootPainter's functionality to extract segmentation metrics they provide a convenient way for users to confirm and report that their trained models have a suitable accuracy.

Although a trend in improvement over time is shown in the segmentation metrics (Fig. 10a,b), there were large fluctuations and for user b on the biopores dataset, with there appearing to be a decrease in the model accuracy towards the end of corrective annotation (Fig. 10b). We expect that the variation in accuracy was caused by both intra‐annotator variation and by the large amount of variation in the quality and difficulty of the images for the network, as can be seen from all plots.

It is important to note that metrics, such as those mentioned already, comparing corrected predictions with predictions are not comparable to completely independent annotations done while blinded to the model predictions. Completely independent annotations include to some degree an amount of irreducible error stemming from observer variation and uncertainty. Corrections, on the other hand, are expected to be of clear errors and may in some cases, therefore, be a more useful measure of the system's performance as assessed by the observer in question.

Although corrective annotation tended to produce models with higher accuracy relative to dense (Table 3), the lack of a statistically significant difference prevents us from coming to a more substantive conclusion about the benefits of corrective over dense annotation. Despite being unable to confirm our second hypothesis, that corrective annotation provides improved accuracy over dense in a limited time period, it is still clear that it will provide many real‐world advantages. The feedback given to the annotator will allow them to better understand the characteristics of the model trained with the annotations applied. They will be able to make a more informed decision about how many images to annotate to train a model to sufficient accuracy for their use case.

Although strong correlation was attained when using the models trained with corrective annotation, they in some cases overestimated (Fig. 7a) or underestimated (Fig. 7b) the objects of interest compared with the manual counts. For the biopores (Fig. 7a), this may be related to the calibration and threshold procedure, which results in biopores below a certain diameter being excluded from the dataset. We inspected the outlier in Fig. 7(b) where RootPainter had overestimated the number of nodules compared with the manual counts. We found that this image (043.jpg) contained many roots that were bunched together more closely than what was typical in the dataset. We suspect this had confused the trained network and could be mitigated by using a consistent and reduced amount of roots per scan, whilst using more of the images for training and annotating for longer to capture more of the variation in the dataset.

In one case, training with corrective annotation failed to produce a model that gave a strong correlation with the manual measurements. This was for the nodules data for user b, where the *R* ^2^ was 0.69. We suspect this was partially due to the limited number of nodules in the training data. Many of the images in the dataset created for training contained no nodules and only included the background. This also meant the annotation was able to finish in less time. We consider this a limitation of the experimental design, as we expect that a larger dataset that allowed for annotating nodules for the full 2 h time period would have provided better insights into the performance of the corrective training procedure.

Fig. 5 shows examples of some of the errors in the nodules dataset. In practice, the annotator would be able to view and correct such errors during training until they had abated. We noticed that many of the nodule errors were smaller false positives, so investigated the effect of filtering out nodules less than a certain size (Notes S6). The substantial improvements in nodule count correlation from 0.69 to 0.93 when using a nodule size threshold can be explained by the removal of smaller false‐positive artefacts. This indicates that the model was producing many small false‐positive predictions, which could also explain some of the overestimation of nodules (Fig. 7b).

The problem with small false positives may have been mitigated with the dense annotations as a larger amount of background examples are added, suppressing more of the false‐positive predictions that arise in the limited training time.

The improvement in *R* ^2^ when removing small nodules may also be due to differences in subjective interpretation of what is a nodule, between the original counter and annotator training the model.

The reduction in *R* ^2^ as dense annotation time increased, shown in nodules b (Fig. 8), was highly unexpected. Although in some cases increasing training data can decrease performance when training CNNs (Nakkiran *et al*., 2019), it is usually the case that the opposite is observed. We suspect these anomalous results are due to the large amount of variation in the success of the dense training procedure, rather than revealing any general relationship between performance and the amount of data used.

As the nodule images are captured in a controlled environment, further improvements to accuracy could be attained by reducing controllable sources of variation and increasing the technical quality of the images. The lighting was also varying for the nodules, with approximately half of the images underexposed. We expect that more consistent lighting conditions would further improve the nodule counting accuracy. Cropping the nodule images manually could also become a time‐consuming bottleneck, which could be avoided by ensuring all the roots and nodules were positioned inside the border and having the placement of the border be fixed in its position in the scanner such that the cropping could be done by removing a fixed amount from each image, which would be trivial to automate.

Fig. 4(a) indicates corrective annotation leads to lower *R* ^2^ in the earlier phases of annotation (e.g. within 60 min). We suspect this is due to dense annotation having an advantage at the start as the user is able to annotate more pixels in less time using dense annotation with no overhead caused by waiting for segmentations from the server. We suspect in many cases that corrective annotation will provide no benefits in terms of efficiency when the model is in the early stages of training, as the user will still have to apply large amounts of annotation to each image whilst being slowed down by the delay in waiting for segmentations. Later in training (e.g. after 1 h 40 min), corrective annotation overtakes dense annotation in terms of mean *R* ^2^ performance (Fig. 4a). We suspect this is due to the advantages of corrective annotation increasing as the model converges, when more of the examples are segmented correctly and do not need adding to the training data as they would provide negligible utility beyond what has already been annotated. Our results show corrective annotation achieves competitive performance with a fraction of the labelled pixels compared with dense annotation (Fig. 9b). These results align with Toneva & Sordoni (2019), who confirmed that a large portion of the training data could be discarded without hurting generalization performance. This view is further supported by theoretical work (Soudry *et al*., 2018) showing in certain cases that networks will learn a maximum‐margin classifier, with some data points being less relevant to the decision boundary.

The corrective training procedure performance had lower SE after 1 h (Fig. 4a), and particularly at the end (Table 3). We conjecture that the corrective annotation strategy stabilized convergence and increased the robustness of the training procedure to the changes in dataset with the fixed hyperparameters by allowing the specific parts of the dataset used in training to be added based on the weaknesses that appear in each specific training run.

In more heterogeneous datasets with many anomalies, we suspect corrective annotation to provide more advantages in comparison with dense annotation, as working through many images to find hard examples will capture more useful training data. A potential limitation of the corrective annotation procedure is the suitability of these annotations when used as a validation set for early stopping, as they are less likely to provide a representative sample, compared with a random selection. Our annotation protocol for corrective annotation involved initially focusing on clear examples (Notes S3), as in preliminary experiments we found corrective annotation did not work effectively at the very start of training. Training start‐up was also found to be a challenge for other systems utilizing interactive training procedures (Gonda *et al*., 2017), indicating future work in this area would be beneficial.

Another possible limitation of corrective annotations is that they are based on the model's weaknesses at a specific point in time. This annotation will likely become less useful as the model drifts away to have different errors from those that were corrected.

One explanation for the consistently strong correlation on the root data compared with biopores and nodules is that the correlation with counts will be more sensitive to small errors than correlation with length. A small pixel‐wise difference can make a large impact on the counts, whereas a pixel erroneously added to the width of a root may have no impact on the length and even pixels added to the end of the root will cause a small difference.

A limitation of the RootPainter software is the hardware requirements for the server. We ran the experiments using two Nvidia RTX 2080 Ti GPUs connected with NVLink. Purchasing such GPUs may be prohibitively expensive for smaller projects, and hosted services such as Paperspace, Amazon Web Services, or Google Cloud may be more affordable. Although model training and data processing can be completed using the client user interface, specialist technical knowledge may still be required to set up the server component of the system. To mitigate the hardware requirements and technical knowledge required for the initial setup, we have prepared an open‐source Jupyter notebook web application (see Data availability section) which is made available via Google Colab and guides a user without specialist technical knowledge through setting up a RootPainter server using a freely available GPU. As part of the online supplementary material we also make a video available showing how to interactively train and use a biopores segmentation model with RootPainter (Video S1).

Although RootPainter automates the annotation process by providing an initial model prediction, the correction process, which involves manually drawing, could still become laborious, especially for larger, more complex images. Interactive segmentation tools, such as Grabber (Bragantini *et al*., 2020), may be complementary and could be investigated in further work to accelerate RootPainter's corrective‐annotation process.

There are a limited number of root traits that can be exported from RootPainter in comparison with other root segmentation software applications, such as faRIA (Narisetti *et al*., 2021). This limitation has been addressed by the addition of a conversion utility, available from the RootPainter extras menu, that enables RootPainter segmentations to be conveniently processed with RhizoVision Explorer (Seethepalli *et al*., 2021), facilitating the extraction of many more traits from the RootPainter segmentations.

In addition to the strong correlations with manual measurements when using corrective annotation, we found the accuracy of the segmentations obtained for biopores, nodules, and roots to indicate that the software would be useful for the intended counting and length measurement tasks (Fig. 6a–c).

The performance of RootPainter on the images not used in the training procedure indicates that it would perform well as a fully automatic system on similar data. Our results are a demonstration that, for many datasets, using RootPainter will make it possible to complete the labelling, training, and data processing within one working day.

## Author contributions

AGS implemented RootPainter and wrote the manuscript with assistance from all authors. AGS, EH, and JP designed the experiment. AGS and EH annotated and collaborated on the design of the study and the introduction. CG and MA captured and prepared the nodules data. NAFO tested the software and annotation protocol during development. DBD and KTK provided supervision and conceptual input. All authors read and approved the final manuscript.

## Supporting information


**Notes S1** Server software set‐up instructions. Instructions on how to set up the RootPainter server.
**Notes S2** Keyboard shortcuts. A table showing the keyboard shortcuts that can be used to speed up the annotation process when using the RootPainter client.
**Notes S3** Corrective training protocol. Instructions on how to train a model with RootPainter using the corrective annotation protocol, as was done in this study.
**Notes S4** Corrective annotation advice. Extra tips on how to execute the corrective training protocol effectively.
**Notes S5** Dense annotation advice. Tips on how to annotate densely, as was done in this study for comparison purposes.
**Notes S6** Nodule threshold plot. A plot showing correlation between automatic and manual nodules counts as a function of a nodule size threshold.

**Video S1** RootPainter biopore model training video. A 42 min video showing the training of a biopore segmentation model using RootPainter with corrective annotation.
Please note: Wiley Blackwell are not responsible for the content or functionality of any Supporting Information supplied by the authors. Any queries (other than missing material) should be directed to the *New Phytologist* Central Office.

## Data Availability

The nodules dataset is available from Smith *et al*. (2020a). The biopores dataset is available from Smith *et al*. (2020b). The roots dataset is available from Smith *et al*. (2019a). The client software installers are available from https://github.com/Abe404/root_painter/releases. The source code for both client and server is available from https://github.com/Abe404/root_painter. The created training datasets and final trained models are available from Smith *et al*. (2020c). The Colab notebook is available at https://colab.research.google.com/drive/104narYAvTBt‐X4QEDrBSOZm_DRaAKHtA?usp=sharing.
