# Supplementary material for: RootPainter: deep learning segmentation of biological images with corrective annotation
Source: New Phytol. 2022 Aug 10;236(2):774–91. doi: 10.1111/nph.18387 (PMC9804377; doi:10.1111/nph.18387)
Supplement: Supplementary file 1 — Notes S1 Server software set‐up instructions. Instructions on how to set up the RootPainter server. Notes S2 Keyboard shortcuts. A table showing the keyboard shortcuts that can be used to speed up the annotation process when using the RootPainter client. Notes S3 Corrective training protocol. Instructions on how to train a model with RootPainter using the corrective annotation protocol, as was done in this study. Notes S4 Corrective annotation advice. Extra tips on how to execute the corrective training protocol effectively. Notes S5 Dense annotation advice. Tips on how to annotate densely, as was done in this study for comparison purposes. Notes S6 Nodule threshold plot. A plot showing correlation between automatic and manual nodules counts as a function of a nodule size threshold. [file NPH-236-774-s002.pdf]

New Phytologist Supporting Information

RootPainter: Deep Learning Segmentation of Biological Images with Corrective Annotation

Abraham George Smith, Eusun Han, Jens Petersen, Niels Alvin Faircloth Olsen, Christian Giese, Miriam Athmann, Dorte Bodin Dresbøll, Kristian Thorup-Kristensen

Article acceptance date: 30 June 2022

Note S1 Server Software Setup Instructions

For our tests we use a client-server architecture and run the client and server components of the system on different computers, using Dropbox to facilitate IO between them. We do not use any Dropbox specific functionality so any service which synchronises a folder between two computers should work. It is also possible to run the client and server on the same computer which will reduce lag and eliminate the need to use a third party service or network drive to sync files. We tested the server component using Python 3.7.5.

1. git clone --branch 0.2.4 `https://github.com/Abe404/root_painter.git`
2. cd root_painter/trainer
3. pip install torch==1.3.1 -f `https://download.pytorch.org/whl/torch_stable.html`
4. pip install -r requirements.txt
5. NOTE: pytorch installation may be more involved as it could require and consideration of the current CUDA version. We have tested using pytorch version 1.3.1 but would expect it to work with more recent versions. See `https://pytorch.org/get-started/previous-versions/` for more details on how to install pytorch.
6. python3 main.py
7. You will be prompted to specify a sync location. For our tests we used Dropbox and a folder named paper_rp_sync so we input ~/Dropbox/paper_rp_sync
8. If the folder doesn't exist then it will be created with the necessary sub folders (datasets, projects, models and instructions).
9. The system will start running and watching for instructions from the client. You must share access to the created folder with the users using your file share service (such as Dropbox) or network drive. The users will then need to input this when they first run the client software.
10. See section *Software Implementation* for further instructions for client software setup.
11. For our tests we ran the RootPainter server inside a tmux session but for more long running use cases a systemd service will likely be more robust. See `https://github.com/torfsen/python-systemd-tutorial` for instructions on creating a systemd service with python.

Note S2 Keyboard Shortcuts

Table 1. Keyboard shortcuts for corrective annotation with RootPainter

Key	Function
Q	foreground brush
W	background brush
Z	undo
Ctrl+Shift+Z	redo
Alt+scroll	Change brush size
(Windows Key/Command key) + click and drag	Pan view
A	show or hide annotation
I	show or hide image
S	show or hide segmentation
Scroll	zoom

Note S3 Corrective Training Protocol

A. Stage 1.

- Start a timer immediately before starting to annotate
- Start training after clicking *Save & Next* for the second annotated image.
- Keep track of how many images you have annotated until you have annotated six images.

- Skip images which do not include clear examples of both classes.
- When images contain clear examples of both classes then label the clear and unambiguous parts of the image.
- Aim to label around 5-10 times as much background as foreground.
- Use a thinner brush to avoid boundaries when labelling the foreground class as these can be ambiguous and time consuming to label.
- After clicking *Save & Next* for the 6th image proceed to stage 2.
- Write down the image number for the 6th annotated image.

B. Stage 2.

- For each image press S to view the segmentation. Instead of labelling everything which is clear, focus on labeling the parts of the image which have clearly been segmented incorrectly, whilst following the corrective annotation advice.
- Once you have proceeded through 10 images since the 6th annotated image then set pre-segment (from the options menu) from 0 to 1. Increasing the pre-segment setting causes the server to create segmentations ahead of time for upcoming images. This allows the user to progress through the images faster but presents a trade-off as they could potentially be out of date as they are segmented with the best model available at the time and not updated. Thus we only increase pre-segment once the user has worked through a few images, as their annotation time speeds up and necessitates the adjustment.
- Once you have proceeded through 20 images since the 6th annotated image then set pre-segment from one to two.
- Once one hour has passed on the timer then take a break for 30 minutes.
- After the 30 minute break then click Start Training again and proceed to annotate as before the break for another hour.
- After the second hour has been completed then stop annotating.
- Leave the network to stop training on its own.

Note S4 Corrective Annotation Advice

- Use a large brush for the background (green) as this makes it quicker label all the false positive regions.
- Focus time and attention on the incorrectly predicted parts of the image
- It is not a problem to label some foreground as foreground which has already been predicted correctly.
- It is also not a problem to label some background as background if it has already been predicted correctly.
- Errors to avoid include labelling a background pixel as foreground or labelling some foreground as background. These should be corrected using the eraser tool.
- It is not a problem to leave small areas unlabelled such as boundaries between foreground and background in the interest of avoiding errors whilst annotating quickly.
- Press I (capital i) to hide and show the image in order to better check the networks segmentation prediction for errors before proceeding to the next image.

Note S5 Dense Annotation Advice

- Set pre-segment (from the options menu) to 10 so that segmentation time does not impact ability to work through the images. Increasing the pre-segment setting causes the server to create segmentations ahead of time for upcoming images. For dense we don't care about the segmentations so by segmenting 10 in advance it means the client software will never stall their progression through the images because the segmentation has not yet loaded.
- Change the background colour from the default transparency level to a transparency level of 8%. This is because altering the brush transparency allows viewing the object of interest through the background annotation.

- Label each image as all background with a single click using the large brush and proceeded to explicitly annotate all objects of interest (using foreground brush) or ambiguous regions (using the eraser brush) before proceeding to the next image.
- Leave ambiguous regions such as boundaries as undefined, rather than labelling them as foreground or background.
- Once the time limit is reached, use the eraser tool to mark areas not yet annotated in the current image as undefined, stop annotating and click *Start Training*.

Note S6 Nodule Threshold Plot

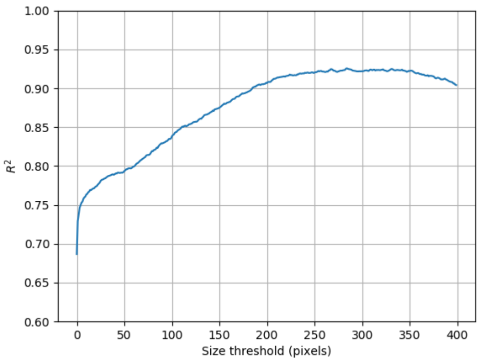

Fig. 1. Correlation between automated and manual nodule counting as a function of size threshold for the automatically detected nodules. The thresholded nodules include only those above the specified area in pixels.

Video S1 RootPainter Biopore Model Training Video

A 42 minute video showing the training of a biopore segmentation model using RootPainter with corrective annotation.

Video

Fig. 2. Embedded Video Placeholder. Video showing the training of a biopores segmentation model.
